# Molecular Hydrogen Attenuates Chronic Inflammation and Delays the Onset of Ultraviolet B-Induced Skin Carcinogenesis in Mice

**DOI:** 10.3390/ijms27020635

**Published:** 2026-01-08

**Authors:** Fumiko Hori, Sayaka Sobue, Chisato Inoue, Yoshiki Murakumo, Masatoshi Ichihara

**Affiliations:** 1 Department of Nursing, College of Life and Health Sciences, Chubu University, Kasugai 487-8501, Japan; 2 Department of Biomedical Sciences, College of Life and Health Sciences, Chubu University, Kasugai 487-8501, Japan; 3 Department of Pathology, School of Medicine, Kitasato University, Sagamihara 252-0374, Japan

**Keywords:** molecular hydrogen (H_2_), ultraviolet B, hairless mouse, cyclobutane pyrimidine dimers, GSH/GSSG, Nrf2, IL-6/STAT3, ERK, JNK, squamous cell carcinoma

## Abstract

Molecular hydrogen (H_2_) exhibits anti-inflammatory and antioxidant properties. However, its role in ultraviolet B (UVB)-induced skin carcinogenesis remains unclear. Male HR-1 hairless mice received continuous H_2_ (2% hydrogen gas inhalation plus hydrogen-rich water (HRW)) or control treatment (normal air plus dehydrogenated water) during chronic dorsal UVB exposure (270 mJ/cm^2^, three times per week, 20 weeks), followed by a 10-week observation period. This protocol was replicated independently. H_2_ exposure consistently delayed the onset of papilloma and reduced cumulative tumor counts in both series, whereas prolonged survival and delayed squamous cell carcinoma (SCC) development each reached statistical significance in only one of the two experimental series. The cyclobutane pyrimidine dimer (CPD) levels remained unchanged, indicating no reduction in DNA photolesions. H_2_ exposure decreased epidermal T-cell infiltration, dermal IL-6 levels, and nuclear phosphorylated STAT3 levels. ERK and JNK phosphorylation levels were decreased. H_2_ preserved the GSH/GSSG ratio following acute UVB exposure and reduced nuclear Nrf2 accumulation during chronic exposure. Epidermal thickness and proliferation markers (Ki-67 and PCNA) were decreased. These findings suggest that continuous H_2_ administration attenuates inflammation-associated early UVB carcinogenesis through modulation of the IL-6/STAT3 and ERK/JNK pathways, supporting its use as a chemopreventive approach.

## 1. Introduction

Squamous cell carcinoma (SCC) represents the second most prevalent form of skin cancer after basal cell carcinoma [1]. While Japan reports approximately 7700 annual cases based on 2016–2018 data [2], the United States has documented approximately 1.8 million cases in 2021 [3], highlighting the substantial public health burden of SCC worldwide. This malignancy predominantly affects elderly populations and constitutes approximately 20% of all skin cancers [1]. Chronic ultraviolet (UV) exposure, particularly ultraviolet B (UVB) radiation (280–320 nm), is the primary etiological factor [4], with sun-exposed regions, such as the head and neck, showing increased susceptibility [1]. UVB radiation induces both direct DNA damage and the generation of reactive oxygen species (ROS), initiating complex inflammatory cascades [4]. Keratinocytes release inflammatory mediators, including interleukin-1 beta (IL-1β), interleukin-6 (IL-6), tumor necrosis factor alpha (TNF-α), and interleukin-8, establishing a pro-inflammatory microenvironment [5] that facilitates all stages of tumorigenesis—from initial proliferation to invasion of transformed cells [6]. The Janus kinase (JAK)/signal transducer and activator of transcription 3 (STAT3) pathway, which is activated by IL-6 signaling, represents a critical molecular link between inflammation and carcinogenesis [7]. Upon activation, STAT3 upregulates multiple target genes, including the anti-apoptotic factor B-cell lymphoma 2 (Bcl-2), cell cycle regulator cyclin D1, and vascular endothelial growth factor (VEGF), collectively promoting cancer cell survival and proliferation [7]. Phosphorylated STAT3 expression correlates positively with tumor invasion depth in human SCC and basal cell carcinoma specimens compared with normal skin [8,9]. These findings suggest that the ROS-STAT3 signaling axis is a central molecular target in UVB-induced skin carcinogenesis.

Molecular hydrogen (H_2_) acts as a bioactive gas that reduces oxidative stress and inflammation, suggesting its therapeutic potential in various pathological conditions [10,11]. H_2_ attenuates both ROS generation and inflammatory cytokine secretion by modulating upstream signaling pathways, including activation of nuclear factor erythroid 2–related factor 2 (Nrf2), inhibition of nuclear factor kappa-light-chain-enhancer of activated B cells (NF-κB) and NLR family pyrin domain containing 3 (NLRP3) inflammasome, and suppression of STAT3 phosphorylation [12]. In the cutaneous tissues of UVB-irradiated rats, treatment with hydrogen-rich saline decreased inflammatory cytokines and oxidative stress markers, thereby mitigating skin damage [13]. Cell culture studies using HaCaT keratinocytes have demonstrated the suppression of UVB-induced ROS generation and lipid peroxidation [14]. Moreover, H_2_ treatment decreases STAT3 phosphorylation and Bcl-2 expression in various organs and tumor models [15,16,17]. As STAT3 activation plays a central role in SCC development, these observations suggest that H_2_ may prevent skin carcinogenesis by disrupting the ROS-inflammation-STAT3 cascade during early UVB exposure.

This study investigated whether H_2_ administration delays SCC onset by suppressing inflammation and STAT3 activation during chronic UVB exposure. We subjected hairless mice to prolonged UVB irradiation while providing continuous H_2_ exposure through environmental enrichment and consumption of hydrogen-rich water (HRW). Our results demonstrated that H_2_ treatment significantly delayed initial lesion formation, with a trend toward delayed SCC onset and extended survival. These results support the use of H_2_ as a feasible preventive intervention targeting the ROS-inflammation-STAT3 cascade in skin carcinogenesis.

## 2. Results

### 2.1. H_2_ Attenuates Acute Cutaneous Responses Induced by Short-Term UVB Irradiation

**Figure 1:**
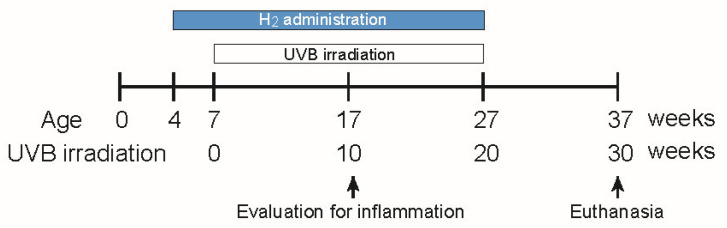
Schematic representation of chronic ultraviolet B (UVB) exposure and molecular hydrogen (H_2_) treatment. Four-week-old HR-1 hairless mice were randomized to H_2_ (2% hydrogen gas inhalation plus hydrogen-rich water (HRW); the dissolved H_2_ concentration ≥ 0.8 mM) or control groups and received dorsal UVB irradiation at 270 mJ/cm^2^ three times per week from 7 to 27 weeks of age. Inflammation and reactive oxygen species (ROS) were evaluated at week 10 of irradiation. Tumors were counted weekly, and the mice were euthanized at 37 weeks of age for histological evaluation.

We examined acute photoinflammatory responses following short-term UVB irradiation under long-term H_2_ administration. Four-week-old mice were randomly assigned to either a control or a H_2_-treated group. The mice in the H_2_-treated group received HRW ad libitum and were housed in chambers containing 2% hydrogen gas. UVB irradiation (270 mJ/cm^2^, three times per week) was initiated when the mice reached 7 weeks of age (Figure 1). Five weeks post-irradiation, marked differences in cutaneous erythema emerged between the groups (Figure S1), suggesting reduced acute photoinflammatory injury similar to that described previously [18].

### 2.2. H_2_ Delays Early Lesion Formation in Tumorigenesis Induced by Chronic UVB Exposure and Shows Trends Toward Postponed SCC Onset with Prolonged Survival

**Figure 2:**
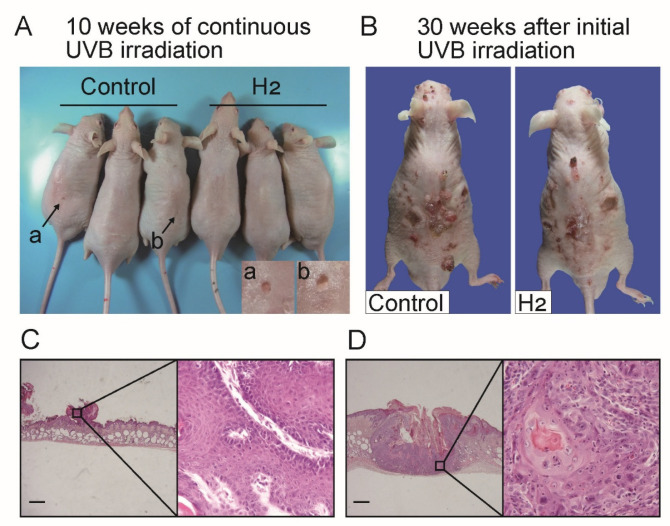
Macroscopic and histological appearances of UVB-induced tumors. Dorsal surface images are shown after 10 weeks of continuous UVB irradiation (**A**) and at 30 weeks after initial exposure (**B**). In (**A**), arrows indicate representative small nodules observed in the control group (Control); the inset shows higher-magnification views of the lesions labeled (**a**,**b**). Compared to the control group (Control), H_2_ administration (H_2_) reduced the number of small nodules and showed a trend toward decreased tumor multiplicity at 30 weeks. Histological examination reveals papilloma characteristics in small nodules that developed after approximately 10 weeks (**C**), whereas invasive squamous cell carcinoma (SCC) is observed in most lesions at 30 weeks (**D**). Scale bars indicate 500 μm.

When hairless mice were exposed to UVB irradiation, papillomas initially developed and subsequently underwent malignant transformation into SCC, as previously reported [19]. In the present study, small tumors of approximately 1 mm in diameter were occasionally observed around 10 weeks after the initiation of UVB irradiation (Figure 2A). Furthermore, 30 weeks after the initial UVB exposure, tumors of various sizes formed on the dorsal skin of the mice (Figure 2B). Histopathological examination revealed that these small tumors exhibited the characteristic features of papillomas (Figure 2C), whereas most tumors observed at 30 weeks displayed the morphological characteristics of SCC (Figure 2D).

To further characterize tumor progression, we examined the histopathological features of tumors formed 30 weeks after the initiation of UVB irradiation. We randomly selected papillomas and SCCs and measured their diameters. As shown in Figure S2, the papillomas with diameters of approximately 1 mm did not exceed 5 mm. In contrast, the diameter of the SCCs ranged from approximately 3 to 10 mm.

**Figure 3:**
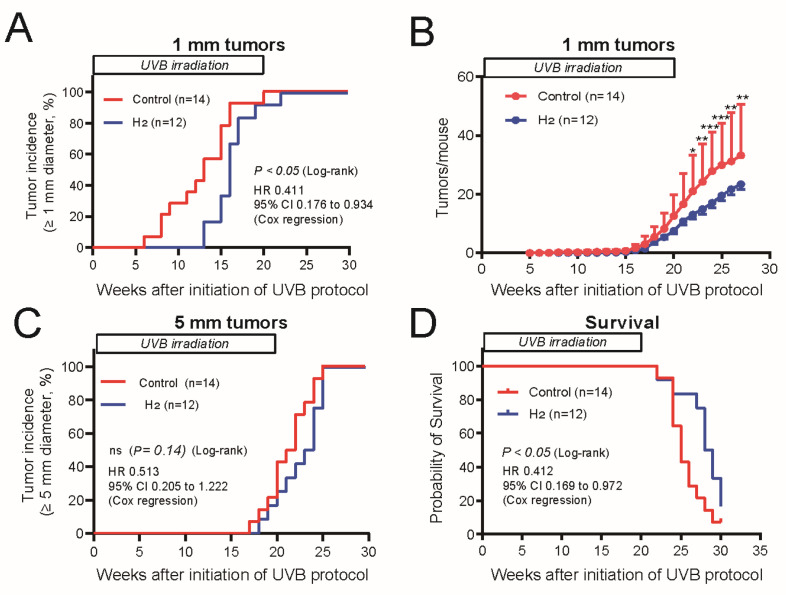
H_2_ delays early lesion formation in tumorigenesis induced by chronic UVB exposure and shows trends toward postponed SCC onset with prolonged survival. (**A**) The percentage of mice with tumors (≥1 mm diameter) in the control (Control) and H_2_-administered (H_2_) mice is plotted against the number of weeks since initiation of the UVB exposure protocol. (**B**) The total number of tumors per mouse in each group is plotted against the number of weeks since initiation of the UVB exposure protocol. (**C**) The percentage of mice with tumors (≥5 mm diameter) in Control and H_2_ groups is plotted against the number of weeks since initiation of the UVB exposure protocol. (**D**) Survival curves for control and H_2_-administered mice. For (**A**, **C** and **D**), the Kaplan–Meier method was used to estimate tumor incidence and survival probability. Differences were assessed by the log-rank test, and Cox proportional hazards regression was used to estimate hazard ratios (HR) and 95% confidence intervals (CI). For (**B**), differences between groups were analyzed using two-way analysis of variance (ANOVA) with Šidák’s post hoc test. * *p* < 0.05; ** *p* < 0.01; *** *p* < 0.001.

Based on these observations, we measured the weekly tumor diameters to investigate the effects of H_2_ on tumor proliferation. Tumors with a diameter of ≥1 mm and ≤2 mm, upon their initial appearance, were classified as papillomas, representing the initial lesions of SCC development, whereas those with a diameter of 5 mm or larger were classified as advanced SCC. We carefully monitored the temporal progression of tumor formation. The appearance of papillomas was significantly delayed in the H_2_-treated group, and a significant reduction in the total number of tumors was observed (Figure 3A,B). The appearance of tumors with a diameter of 5 mm or larger was used as an indicator of malignant progression to SCC. Although no statistically significant differences were observed, the H_2_-treated group showed a clear trend toward delayed carcinogenesis (Figure 3C). In the survival analysis, hairless mice were monitored for 30 weeks after irradiation, and the survival rate was significantly prolonged in the H_2_-administered group compared to that in the control group (Figure 3D).

To confirm the reproducibility of the tumor-suppressive effect of H_2_ on UVB-induced skin carcinogenesis, the experiment was repeated using the same protocol. In this experimental series, H_2_ significantly delayed papilloma onset (Figure S3A), reduced the total number of papillomas (Figure S3B), and significantly delayed the onset of SCC (Figure S3C), with a trend toward extended survival (*p* = 0.081) (Figure S3D). Pathological examination was performed on tumor tissues collected 30 weeks after the initiation of UVB irradiation. In the control group, tumor enlargement and confluence after 28 weeks resulted in a reduced number of tumors available for pathological evaluation; however, this reduction was not statistically significant. H_2_ administration did not modify the histological appearance or characteristics of the carcinomas induced by UVB irradiation (Table 1).

### 2.3. H_2_ Does Not Suppress the Formation of UVB-Induced Pyrimidine Dimers in the Skin

We hypothesized that H_2_ administration influences the development of UVB-induced skin cancer by affecting pyrimidine dimer formation, which is the primary DNA lesion induced by UVB irradiation in the skin. Therefore, we examined cyclobutane pyrimidine dimer (CPD) formation in hairless mice pretreated with H_2_ for 3 weeks before UVB irradiation and compared these results with untreated controls (Figure S4). No difference in CPD formation was observed between the H_2_-administered and control groups, indicating that the protective effects of H_2_ occurred through mechanisms independent of preventing DNA damage.

### 2.4. H_2_ Attenuates UVB-Induced Inflammation in the Skin

**Figure 4:**
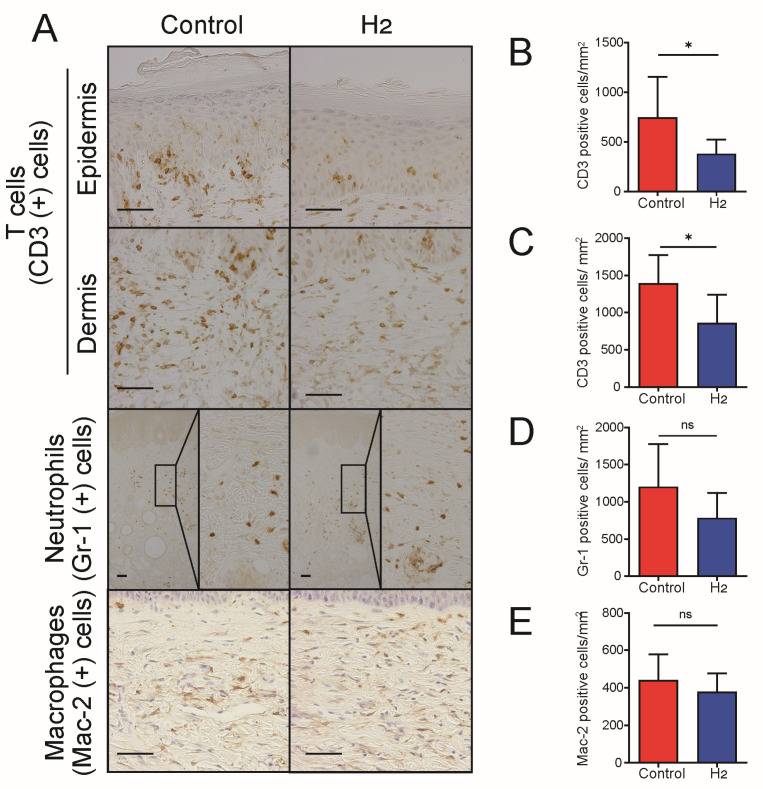
H_2_ treatment decreases T-cell infiltration in skin following chronic UVB exposure. (**A**) Immunohistochemical staining for CD3^+^ T cells, Gr-1^+^ neutrophils, and Mac-2^+^ macrophages in the epidermal and dermal compartments. Quantitative analysis of CD3^+^ cell numbers in the epidermis (**B**) and dermis (**C**) demonstrates a significant reduction following H_2_ treatment. Quantitative analysis of Gr-1^+^ neutrophils (**D**) and Mac-2^+^ macrophages (**E**) reveals no significant change in neutrophil infiltration and a modest decrease in macrophage accumulation. Data are presented as mean ± SD (*n* = 7). * *p* < 0.05; ns indicates not significant. Scale bars indicate 50 μm.

A well-established biological effect of H_2_ is the reduction in inflammation [11,20]. Given that inflammation plays a crucial role in the progression of UVB-induced skin cancer [5,21], we investigated the extent to which H_2_ reduces UVB irradiation-induced inflammation. Skin samples from the dorsal region of hairless mice were collected 10 weeks after the initiation of UVB irradiation, and the distribution of inflammatory cells was examined by immunohistochemical staining (Figure 4A). In hairless mice treated with H_2_, a significant reduction in T cells was observed in both the epidermis and dermis compared to control mice (Figure 4B,C). Although no statistically significant differences were observed in the macrophage and neutrophil populations, a clear trend toward reduction was noted with H_2_ administration, suggesting an overall reduction in chronic inflammation (Figure 4D,E).

Subsequently, real-time PCR was used to quantify the expression of inflammatory mediators in the dorsal skin of hairless mice 10 weeks after UVB irradiation. Expression levels of four key inflammatory mediators—TNF-α, IL-1β, IL-6, and prostaglandin-endoperoxide synthase 2 (Ptgs2)—in the skin were compared between H_2_-treated and untreated groups. However, no significant reduction was observed in any of these markers. This unexpected finding may be attributed to mechanisms such as cytokine resistance mediated by epigenetic modifications following repeated stimulation [22] or the activation of redundant pathways [23] that maintain chronic inflammation despite H_2_ treatment (Figure S5A–D).

To better understand the anti-inflammatory effects of H_2_, we examined skin samples collected 8 h after a single UVB irradiation, and confirmed that H_2_ suppressed the expression of these inflammatory mediators during the acute phase. A significant reduction in IL-6 expression and a clear trend toward reduced IL-1β were observed, findings consistent with previous reports demonstrating the anti-inflammatory effects of H_2_ (Figure S5E–H).

### 2.5. H_2_ Attenuates STAT3 Activation and IL-6 Production in Long-Term UVB-Irradiated Skin

**Figure 5:**
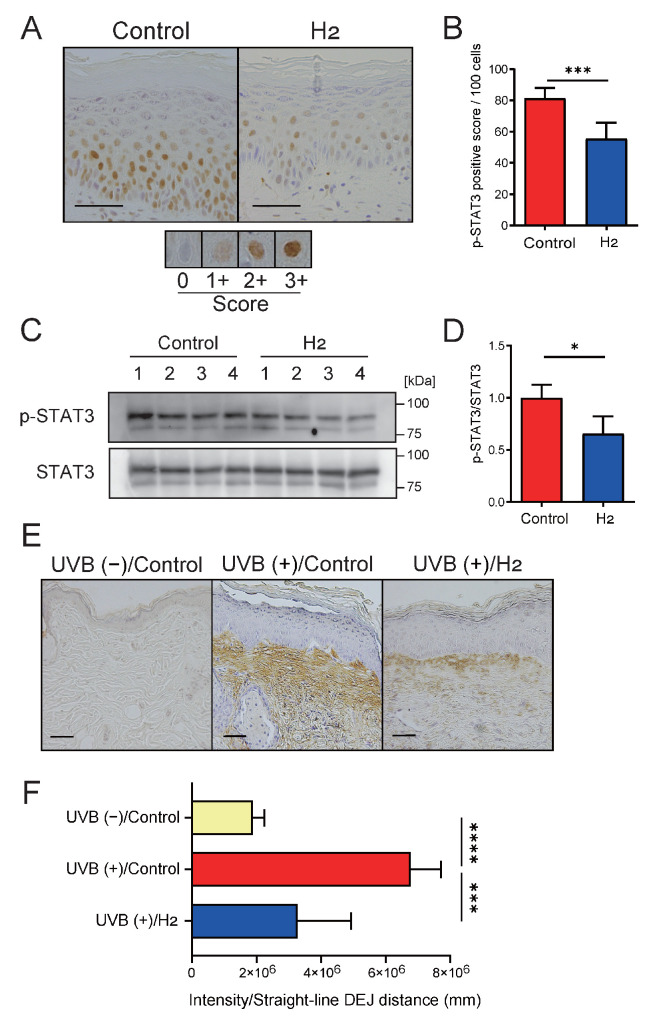
H_2_ treatment suppresses signal transducer and activator of transcription 3 (STAT3) activation and restricts the spatial distribution of interleukin-6 (IL-6). (**A**) Immunohistochemistry of phospho-STAT3 (p-STAT3) nuclear localization. Scoring criteria for immunostaining are shown in the figure. (**B**) Quantification showing reduced p-STAT3-positive nuclei with H_2_ treatment (*n* = 7). (**C**) Immunoblot analysis of p-STAT3 and total STAT3 levels. (**D**) Densitometric quantification demonstrating decreased p-STAT3/STAT3 ratio with H_2_. (**E**) IL-6 immunohistochemistry of UVB (−)/Control, UVB (+)/Control, and UVB (+)/H_2_-treated skin. UVB exposure induced a broad subepidermal IL-6–positive region, which was markedly reduced by H_2_ treatment. (**F**) Quantitative analysis of IL-6 staining intensity beneath the dermal–epidermal junction (DEJ) showed that H_2_ treatment significantly attenuated the UVB-induced increase in IL-6 (*n* = 4–7). Scale bars indicate 50 μm. Data are presented as mean ± SD. * *p* < 0.05; *** *p* < 0.001; **** *p* < 0.0001.

STAT3 activation is strongly implicated in the development of inflammation-associated UVB-induced skin cancer [24]. To investigate the changes in STAT3 activation in the skin following H_2_ administration, we examined the UVB-induced nuclear translocation of phosphorylated STAT3 in dorsal skin samples collected 10 weeks after UVB irradiation by immunohistochemical staining (Figure 5A,B). Quantitative comparison of nuclear-positive cells revealed a significant reduction in STAT3 activation in H_2_-treated hairless mice compared with controls. Additionally, Western blot analysis independently confirmed reduced STAT3 activation in the skin of mice treated with H_2_ (Figure 5C,D).

Next, we examined the expression of IL-6, a major upstream regulator of STAT3 activation, in the skin. Figure 5E shows extensive IL-6 immunoreactivity throughout the subcutaneous tissue in the control samples, consistent with previous reports [25]. H_2_ treatment significantly reduced IL-6 immunoreactivity in localized regions of subcutaneous tissue. A quantitative comparison of the IL-6-positive staining areas below the dermal-epidermal junction revealed a significant reduction in the H_2_-treated group (Figure 5F). Although these changes were not confirmed at the mRNA level, reduced IL-6 protein production in chronically UVB-irradiated skin after H_2_ administration likely weakened STAT3 activation. Alternatively, H_2_ may directly weaken STAT3 activation induced by stimuli other than IL-6, and both possibilities remain plausible.

### 2.6. H_2_ Attenuates Extracellular Signal-Regulated Kinase (ERK) and c-Jun N-Terminal Kinase (JNK) Signaling in Long-Term UVB-Irradiated Skin

**Figure 6:**
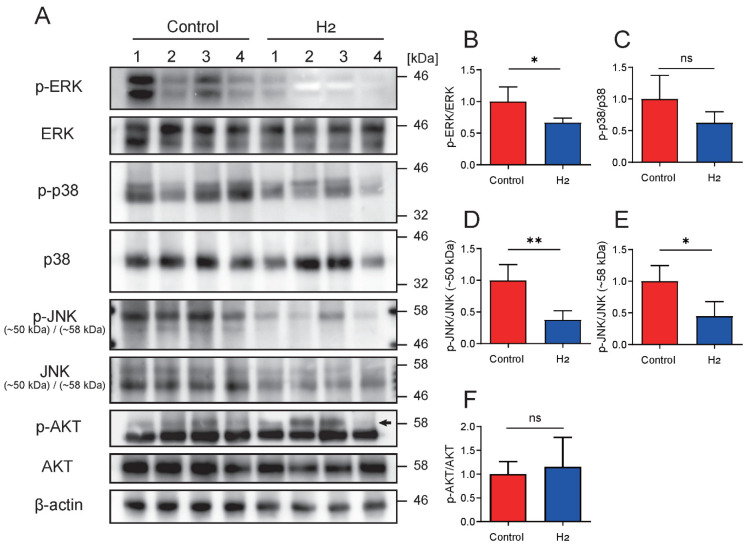
H_2_ selectively inhibits extracellular signal-regulated kinase (ERK) and c-Jun N-terminal kinase (JNK) phosphorylation without affecting p38 or protein kinase B (AKT). (**A**) Representative immunoblots of phospho-ERK (p-ERK)/total ERK, phospho-p38 (p-p38)/total p38, phospho-JNK (p-JNK)/total JNK (two bands at ~50 kDa and ~58 kDa), and phospho-AKT (p-AKT)/total AKT, with β-actin as the loading control. Molecular weight markers (kDa) are shown; the arrow indicates the AKT1 band used for quantifying p-AKT. (**B**–**F**) Densitometric quantification of phospho/total ratios for ERK (**B**), p38 (**C**), JNK (~50 kDa) (**D**), JNK (~58 kDa) (**E**), and AKT (**F**). H_2_ treatment significantly decreased p-ERK and p-JNK levels, whereas p-p38 and p-AKT remained unchanged. Data are presented as mean ± SD. * *p* < 0.05; ** *p* < 0.01; ns indicates not significant.

Given that H_2_ administration was observed to reduce inflammation and weaken specific intracellular signaling pathways [11,26,27], we extended our analysis beyond STAT3 to examine the activation status of other key signaling molecules. Specifically, we assessed the phosphorylation levels of ERK, p38 mitogen-activated protein kinase (p38), JNK, and protein kinase B (AKT) in skin samples collected 10 weeks after UVB irradiation by Western blot (Figure 6A–F). Quantitative analysis of band intensities confirmed that the activation of ERK and JNK was significantly reduced in the H_2_-treated group, whereas the phosphorylation levels of p38 and AKT remained unchanged.

### 2.7. H_2_ Alleviates Skin Thickening and Inhibits Cellular Proliferation in Chronically UVB-Irradiated Skin

**Figure 7:**
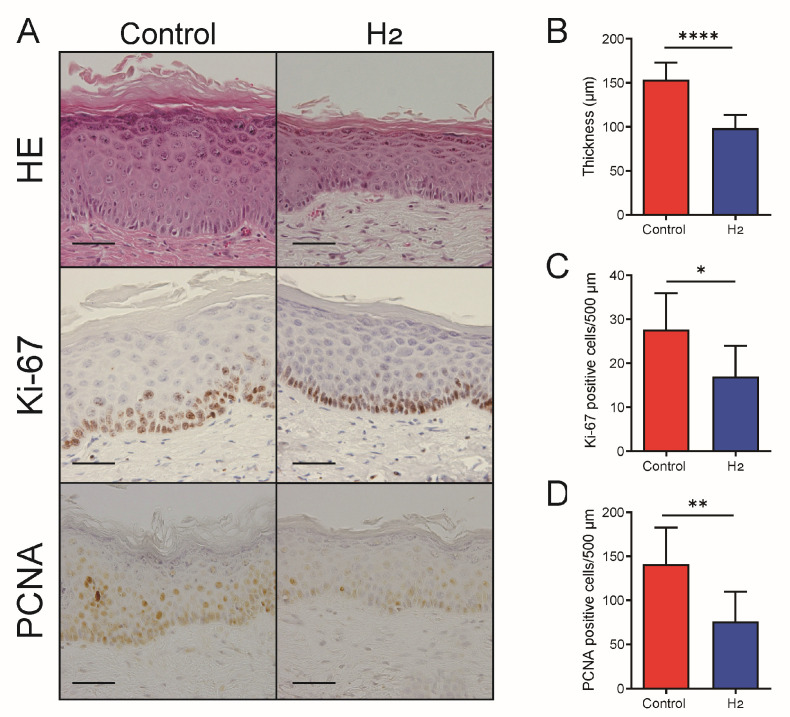
H_2_ attenuates epidermal thickening and cellular proliferation following chronic exposure to UVB radiation. (**A**) Representative images of hematoxylin and eosin (HE), Ki-67, and proliferating cell nuclear antigen (PCNA) immunostaining showing decreased epidermal thickness and reduced proliferating cell numbers in H_2_-treated samples. Scale bars indicate 30 μm. (**B**) Quantification of epidermal thickness. (**C**) Quantification of Ki-67-positive cells per 500 µm. (**D**) Quantification of PCNA-positive cells per 500 µm. H_2_ treatment significantly reduced epidermal thickness and proliferative index compared with control. Data are presented as mean ± SD (*n* = 7). * *p* < 0.05; ** *p* < 0.01; **** *p* < 0.0001.

We examined epidermal thickness and cellular proliferation in skin exposed to UVB irradiation for 10 weeks. Reflecting the observed attenuation of intracellular signaling pathways, the H_2_-treated group showed a significant reduction in epidermal thickness compared with the control group (Figure 7A,B). Moreover, the number of proliferating cells, as assessed by immunostaining for Ki-67 (Figure 7A,C) and proliferating cell nuclear antigen (PCNA) (Figure 7A,D), was significantly decreased in the H_2_-treated group.

### 2.8. H_2_ Suppresses ROS Production Following Both Acute and Chronic UVB Exposure

**Figure 8:**
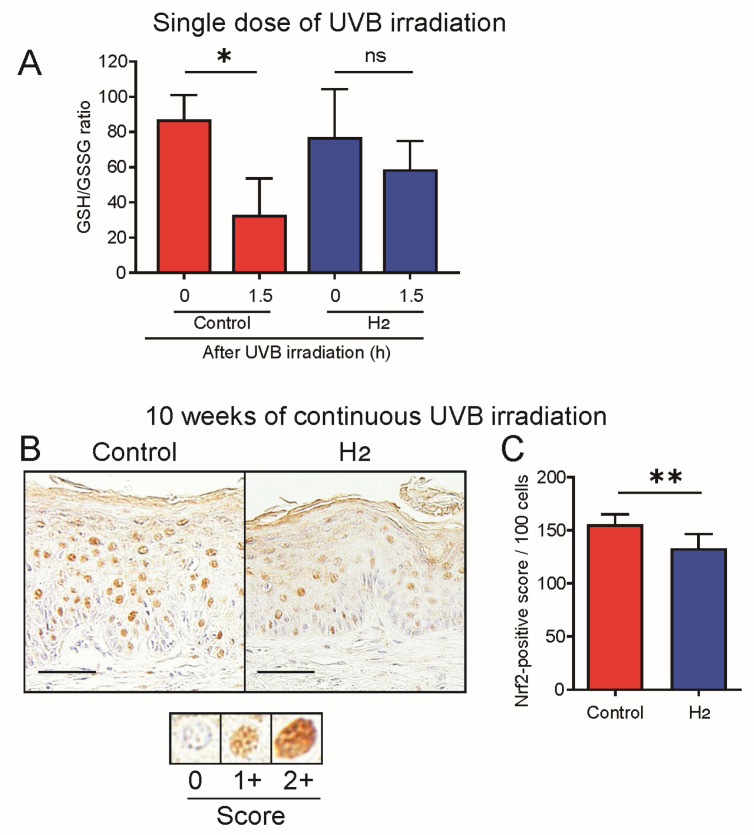
H_2_ reduces oxidative stress markers in acute and chronic UVB-induced skin damage. (**A**) Reduced glutathione/glutathione disulfide (GSH/GSSG) ratio in the acute UVB model. Single-dose UVB irradiation decreased the GSH/GSSG ratio at 1.5 h in control mice, whereas H_2_ pre-treatment preserved the ratio (*n* = 5–6). (**B**) Representative immunohistochemistry images of nuclear Nrf2 in the chronic UVB model (10 weeks). (**C**) Quantification of nuclear Nrf2 (nuclear factor erythroid 2–related factor 2)-positive scores per 100 cells. H_2_ treatment decreased nuclear Nrf2 accumulation, indicating reduced ROS-induced activation (*n* = 6–7). Scale bars indicate 50 μm. Data represent mean ± SD. * *p* < 0.05; ** *p* < 0.01; ns indicates not significant.

In various animal disease models, H_2_ administration consistently reduced ROS levels. Therefore, in this study, we investigated the effects of H_2_ on ROS production in a UVB-induced skin cancer model. Initially, we examined the effects of H_2_ on ROS levels in the skin exposed to a single UVB irradiation event after three weeks of H_2_ pre-administration, using the reduced glutathione/glutathione disulfide (GSH/GSSG) ratio as a quantitative measure of oxidative stress. In the control group without H_2_ administration, a significant decrease in the GSH/GSSG ratio, attributed to ROS production, was observed following UVB irradiation. In contrast, no significant decrease in the GSH/GSSG ratio was observed in the H_2_-administered group, confirming the inhibitory effect of H_2_ on UVB-induced ROS production in the skin (Figure 8A).

To evaluate the effect of H_2_ on ROS production in skin subjected to chronic UVB exposure, we first compared malondialdehyde levels as a marker of lipid peroxidation. However, no significant difference was observed between the H_2_-treated and control groups, most likely due to the long-term accumulation of lipid oxidation products resulting from the high levels of ROS generated by weekly UVB irradiation over the 10 weeks.

Because the nuclear translocation of Nrf2, which is activated by ROS, is a reversible process that declines within a short period of approximately one day [28,29], we evaluated the oxidative stress status using the nuclear translocation of Nrf2 as a dynamic indicator to assess short-term ROS turnover. As shown in Figure 8B, the nuclear translocation of Nrf2 was visibly reduced in the H_2_-treated group compared to that in the control group. Quantitative analysis of the nuclear translocation signal confirmed that nuclear Nrf2 accumulation was significantly reduced in the H_2_-treated group (Figure 8C).

## 3. Discussion

Our study demonstrated that continuous administration of H_2_, via HRW consumption and hydrogen gas inhalation, for approximately six months reduced the cumulative number of UVB-induced skin tumors in hairless mice. This treatment significantly delayed the onset of early lesions, reduced tumor multiplicity, and extended survival. Although SCC onset was not significantly delayed in the initial cohort, a significant delay was observed in the independent replicates. Although researchers have previously combined H_2_ administration with UV irradiation in both human and mouse models, these studies have concentrated on acute responses following brief UV exposure [18]. They measured immediate outcomes such as ROS reduction or inflammatory cytokine levels [30,31,32] without examining carcinogenesis. Long-term investigations remain limited to two reports: Kiyoi et al. found that six weeks of H_2_ treatment suppressed ultraviolet A (UVA)-induced epidermal hyperplasia and melanin production in mice [33], whereas Kato et al. documented wrinkle reduction following three months of hydrogen baths in humans [34].

Beyond keratinocyte-dominant models, H_2_ also exerts cytoprotective effects in human melanocytes. For instance, Fang et al. demonstrated that H_2_ reduced oxidative stress and protected human melanocytes through Nrf2 signaling activation [35]. Further, topical HRW has demonstrated improvements in objective skin parameters including pigmentation-related endpoints in a pilot human study [36]. These observations suggest that H_2_ can modulate redox and pigment-associated pathways under oxidative stress conditions relevant to UV-driven skin biology. Although our current work addresses UVB-induced keratinocyte carcinogenesis, the common involvement of oxidative stress and Nrf2-related signaling in both keratinocytes and melanocytes warrants future studies using melanoma-relevant models to directly test whether H_2_ impacts melanocyte transformation or melanoma initiation/progression.

Several animal studies have demonstrated that H_2_ administration modifies carcinogenesis in various disease models, beyond UV-induced skin cancer. In a mouse model of non-alcoholic steatohepatitis (NASH) progression to hepatocellular carcinoma, eight weeks of HRW consumption significantly reduced liver tumor incidence and nodule size compared to controls [37]. Similarly, HRW consumption suppresses early tumor-promoting events in the kidney in a rat model of iron nitrilotriacetate (Fe-NTA)-induced chemical carcinogenesis [38]. Furthermore, pre-irradiation administration of hydrogen-rich saline significantly decreased the incidence of thymic lymphoma in a radiation-induced carcinogenesis mouse model compared to that in controls [39]. Collectively, these studies suggest that the anti-carcinogenic effects of H_2_ are primarily attributed to its antioxidant activity and reduced oxidative stress.

In our study, the administration of H_2_ to UVB-irradiated hairless mice delayed the onset of early SCC lesions; however, the distribution of histological patterns, differentiation grade, and invasion depth did not differ significantly at 30 weeks post-irradiation. Under our dosing conditions, UVB-induced CPD formation did not differ between groups. By contrast, topical application of H(H_2_O)m, an “atomic hydrogen surrounded by water molecules” formulation, before UV irradiation suppressed DNA damage, assessed by UV-induced erythema and thymine dimer formation, in human skin [18]. H(H_2_O)m administration may provide stronger protection to the genome from DNA damage than H_2_ administration via inhalation or free drinking. Furthermore, intermittent H_2_ inhalation reduced the formation of 8-hydroxy-2′-deoxyguanosine (8-OHdG) (a marker of oxidative DNA damage) induced by UVA irradiation [33]. 8-OHdG is a biomarker of DNA oxidative damage caused by ROS [40], although it was not measured in our study. Considering the oxidative stress-reducing effects of H_2_ administration, similar results are expected in our hairless mouse model. Collectively, H_2_ administered as an inhaled gas or HRW likely does not directly interfere with UV photoproduct formation, mutation acquisition, or clonal selection. Instead, it may influence tumor formation by modulating the tumor microenvironment, including inflammatory responses and intracellular signaling, while reducing oxidative stress.

H_2_ does not undergo conventional enzymatic metabolism; instead, its in vivo fate is characterized by rapid systemic diffusion, elimination, and partial tissue consumption. After ingesting HRW, breath H_2_ concentration increases within minutes and returns to the baseline within approximately 45–60 min [41]. A human mass-balance study demonstrated that ~59% of ingested H_2_ is eliminated via exhalation and ~0.1% is eliminated via the body surface, whereas the remaining ~40% is unrecovered and presumably retained in tissues or consumed under oxidative conditions [42].

Consistent with partial in vivo consumption, whole-body H_2_ consumption during low-level inhalation (160 ppm) has been quantified at approximately 0.7 μmol/min/m^2^ body surface area [43]. Pharmacokinetic profiles further vary with the administration route. In rodents, oral HRW produces a rapid but transient increase in the H_2_ concentration in the liver and venous blood, whereas arterial levels remain substantially lower; in contrast, inhalation elevates H_2_ concentration similarly in both venous and arterial blood [44]. Supporting this route-dependent distribution, a porcine study demonstrated that jejunal delivery of hydrogen-rich solution increased portal venous H_2_ concentration whereas carotid arterial levels remained undetectable, indicating rapid first-pass hepatic removal and/or pulmonary elimination before systemic distribution [45]. Tissue distribution of H_2_ is further complicated by an intriguing observation: hepatic glycogen reportedly retains H_2_ after oral administration, suggesting that the liver may function as a transient reservoir [46].

These pharmacokinetic features indicate that HRW provides transient bolus exposure, whereas inhalation results in more sustained systemic availability. Consequently, repeated administration is a rational approach for long-term modulation of inflammation-associated signaling [42,44]. Prior in vivo studies further suggest that efficacy depends on the administration regimen; for instance, continuous 2% hydrogen gas inhalation produced only marginal effects, whereas intermittent 2% hydrogen gas exposure and HRW conferred clearer protection, consistent with a signal-modulating mechanism benefitting from repeated transient H_2_ elevations [41]. Based on these observations, we employed a combined protocol of continuous 2% hydrogen gas inhalation to maintain baseline availability plus ad libitum HRW to superimpose intermittent boluses, thereby compensating for rapid clearance and ensuring sustained H_2_ availability throughout the chronic UVB exposure period [42,44].

H_2_ administration suppresses ROS production in animal disease models. In this study, we evaluated ROS suppression in the skin and found that, in hairless mice exposed to UVB for 10 weeks, H_2_ administration significantly reduced the nuclear localization of Nrf2. A single UVB exposure induced oxidative stress in hairless mouse skin, and H_2_ administration effectively reduced this oxidative stress. In addition to the direct scavenging of hydroxyl radicals and peroxynitrite [10], H_2_ exerts antioxidant effects through Nrf2 activation via hormetic mechanisms [47]. Studies in Nrf2-knockout mice have demonstrated that Nrf2 activation mediates the anti-inflammatory effects of H_2_ [48], establishing Nrf2 as a key molecule in the antioxidant and anti-inflammatory actions of H_2_ [49]. Recently, Fe-porphyrin was identified as a molecular target of H_2_ [50], and Fe(II)-heme oxidation by hydroxyl radicals yielded Fe(III)–OH adducts that can react with H_2_ to form Fe(III)–H hydride species. These adducts may oxidize cysteine residues in the Keap1 sensor domain, thereby releasing and activating Nrf2 [51].

Recent evidence further suggests that H_2_ can interact with redox-active metalloproteins beyond its proposed reactivity toward highly reactive oxidants such as hydroxyl radicals and peroxynitrite. In particular, the Rieske iron–sulfur protein (RISP), a catalytic subunit of mitochondrial electron transport chain complex III, has been identified as a primary molecular target of H_2_ [52]. H_2_ activates mitochondrial Lon peptidase 1 (LONP1) and promotes RISP degradation, leading to induction of the mitochondrial unfolded protein response (UPRmt). Structural analyses have revealed that the [2Fe–2S] cluster of the Rieske center is coordinated by two cysteine and two histidine residues; notably, the histidine ligands are solvent-exposed, potentially allowing small molecules to access the redox cofactor. These findings, together with evidence that Fe-porphyrin can participate in H_2_-responsive redox chemistry, support a model wherein H_2_ influences carcinogenesis by modulating metal-centered redox chemistry and oxidant-sensitive signaling rather than conventional receptor binding. In UVB-induced skin carcinogenesis, such redox modulation may reduce oxidative stress and attenuate downstream pro-inflammatory and pro-proliferative pathways, including IL-6/STAT3 and ERK/JNK, thereby preferentially affecting tumor promotion rather than UV photoproduct-driven initiation.

Although H_2_ can induce rapid Nrf2 activation when cells encounter ROS in H_2_-rich environments, prolonged ROS production, combined with sustained H_2_ exposure, likely establishes a different equilibrium. Consistent with this, human adipose tissue cultured in a H_2_-enriched medium showed decreased Nrf2 expression [53]. Thus, the reduced Nrf2 activity observed in H_2_-treated skin likely reflects an equilibrium between UV-induced ROS production and H_2_/Nrf2-mediated scavenging, resulting in efficient ROS removal and a reduced need for Nrf2 activation.

Our observations indicated that UVB simultaneously generated ROS and caused direct DNA photodamage in the skin. These ROS establish a chronic inflammatory microenvironment by inducing IL-1β, IL-6, and TNF-α production from keratinocytes and immune cells. This inflammatory response drives SCC onset and progression [5]. The delayed onset of UVB-induced SCC in our experiments likely results from H_2_-mediated reduction of ROS, which diminishes inflammation, or from the direct anti-inflammatory properties of H_2_ that prevent initial lesion formation. Consistent with this framework, antioxidant interventions—such as polyphenol-rich blackberry extract, which lowers cyclooxygenase-2 (COX-2), inducible nitric oxide synthase (iNOS), prostaglandin E_2_ (PGE_2_), and inhibits mitogen-activated protein kinases (MAPK) (ERK, JNK, p38) and NF-κB/p65—mitigate UV-induced skin injury [54]. Similarly, in fine particulate matter (PM2.5)-induced pneumonia models, N-acetylcysteine (NAC) administration, which replenishes intracellular glutathione, exerts potent antioxidant effects, reduces ROS-dependent neutrophil and monocyte airway infiltration, and alleviates inflammation [55]. Excessive ROS activates the NF-κB transcription factor, inducing the expression of IL-1β, TNF-α, and adhesion molecules [56]. UVB radiation strongly activates ERK/JNK in keratinocytes, directly promoting tumorigenesis by enhancing cell proliferation, evading apoptosis, and activating activator protein-1 (AP-1) [57]. Additionally, ROS stimulates both the MAPK pathway [58] and the NLRP3 inflammasome [59], thereby amplifying the production of inflammatory mediators. Therefore, we hypothesized that H_2_-mediated reduction of ROS attenuates inflammation and intracellular signaling in UV-irradiated epithelia, thereby reducing epithelial hyperplasia and proliferation. Consequently, H_2_ likely delays tumor development.

H_2_ administration significantly reduced T-cell infiltration into the epidermis and dermis after chronic UVB exposure, restricted the distribution of IL-6 protein to the subcutaneous tissue, and decreased STAT3 nuclear translocation. Since aberrant STAT3 activation is widely implicated in epithelial tumorigenesis, including cutaneous SCC, and its constitutive phosphorylation upregulates pro-survival and pro-proliferative genes, these changes are mechanistically consistent with delayed tumor development [60]. H_2_ appears to suppress UV-induced chronic inflammation through two mechanisms: a reduction in oxidative stress and the inhibition of inflammation-associated signaling pathways, specifically the IL-6/STAT3 and ERK/JNK cascades. Collectively, these effects impair the early stages of SCC development triggered by UV-induced chronic inflammation, consequently delaying papilloma formation and reducing tumor multiplicity. Although the mRNA expression of inflammatory cytokines showed no significant alterations during the chronic phase, a marked suppression of IL-6 was observed 8 h after acute UVB exposure. This suggests that the cumulative inhibition of acute responses manifests as changes in protein levels during chronic inflammation.

Macrophages represent another immune compartment capable of shaping the UV-exposed skin microenvironment. In this study, infiltration of Mac-2+ macrophages remained low and showed no significant difference between the control and H_2_ groups, indicating that H_2_ did not substantially alter the total macrophage abundance under our experimental conditions. However, macrophage polarization and function can change independently of total cell numbers; indeed, recent studies in cutaneous wound models indicate that hydrogen gas inhalation promotes early M2-like polarization and attenuates inflammation [61,62]. H_2_ may thus influence macrophage phenotype and cytokine output rather than recruitment. Future investigations should characterize macrophage subsets (such as iNOS/CD86 versus Arg1/CD206) using flow cytometry or multiplex immunostaining to determine whether macrophage-derived cytokines contribute to IL-6/STAT3 signaling during UVB-driven tumor promotion. Beyond photocarcinogenesis, these immunomodulatory properties may also be relevant to inflammatory dermatoses such as atopic dermatitis, acne, or psoriasis, though further controlled studies are warranted.

H_2_ administration delayed the onset of UVB-induced initial SCC lesions by a median of 3 weeks in two independent studies. Studies comparing mouse and human aging have indicated that tumor formation progresses 45-fold faster in 17-week-old mice than in humans [63], implying that a 3-week delay in mice corresponds to approximately 2 years and 7 months in humans. Although cross-species scaling of aging and tumorigenesis is approximate, these findings suggest that H_2_ administration may help reduce the incidence of age-associated diseases at the population level.

Species-specific differences characterize the early lesions of UV-induced skin tumors: mice typically develop papillomas [64], whereas humans present with precancerous intraepithelial lesions, such as actinic keratosis (AK) [65] and Bowen’s disease (also known as squamous cell carcinoma in situ) [66]. These differences necessitate careful interpretation when extrapolating data from mice to human pathologies. Multiple interacting factors likely underlie these variations: differences in skin architecture [67], cells of origin [68,69], UV exposure protocols [70], genetic backgrounds [71], and the spectrum and function of early driver mutations, with humans showing early TP53 and NOTCH loss-of-function linked to differentiation defects [72], whereas murine UVB models prominently display epidermal hyperplasia and increased proliferation [73]. Accordingly, mouse models that better recapitulate the human UV-induced SCC—aligning UV spectrum, genetic background, and target cells—should enable more accurate assessment of the effects of H_2_ on human skin SCC development.

This study had several limitations. First, H_2_ was administered as a combined regimen (continuous 2% inhalation plus ad libitum HRW), and arms receiving inhalation alone, HRW alone, or intermittent inhalation schedules were not included. Further, we did not directly quantify blood, skin, or tissue H_2_ concentrations. The administered dose was therefore operationally defined by the chamber H_2_ concentration (2 vol%) and the dissolved H_2_ concentration in drinking water (≥0.8 mM). Prior pharmacokinetic studies have shown that HRW produces a rapid but transient rise in tissue H_2_, whereas inhalation yields more uniform blood levels [44,74]. As tissue H_2_ levels may vary with the administration route used, direct measurement of H_2_ concentrations in target tissues is essential in future work to delineate route- and pattern-specific exposure profiles and to link local H_2_ availability to modulation of IL-6/STAT3 and ERK/JNK signaling. Second, our evaluation focused primarily on early tumor development, leaving the effects on established SCC proliferation, invasion, metastasis, and the tumor immune microenvironment unexplored. Previous cell line studies have suggested that H_2_ supplementation promotes cell proliferation when mitochondrial function is high [75]. Recent work has identified RISP as a primary target of H_2_ and proposed that RISP loss with subsequent UPRmt induction underlies the pleiotropic, context-dependent effects of H_2_ [52]. This provides a potential mechanistic basis for paradoxical observations; future studies should therefore evaluate mitochondrial proteostasis, UPRmt markers, and tumor growth dynamics in UVB-exposed skin under continuous H_2_ administration. Third, the delay in SCC onset did not reach statistical significance, and the survival extension remained borderline in the reproducibility experiments. This may depend on statistical power or the definitions of events. Fourth, this model was restricted to UVB exposure alone in male HR-1 hairless mice, limiting its external validity to human UVA/UVB mixed exposure and carcinogenesis through AK/Bowen disease. Fifth, the observed suppression of IL-6/STAT3 and ERK/JNK represents correlational findings, and causal relationships through inhibitors or genetic interventions remain unverified. Sixth, although DNA photoproducts (CPDs) remained unchanged, we did not perform a comprehensive analysis of oxidative DNA damage or mutational burden. Based on these considerations, future studies should address route-specific administration with exposure measurements, the effects on established tumors, reproduction under UVA-inclusive conditions in females and other strains, and mechanistic validation using interventional approaches.

## 4. Materials and Methods

### 4.1. Mice

Four-week-old male hairless mice (Hos:HR-1) were purchased from Japan SLC (Hamamatsu, Japan). The mice were housed in plastic cages under controlled conditions (22 °C ± 2 °C; 50% ± 10% relative humidity) with a 12 h light–dark cycle (lights on at 08:00), and fed a standard rodent chow diet (CRF-1; Oriental Yeast Co., Ltd., Tokyo, Japan) ad libitum. As shown in Figure 1, the mice received H_2_ treatment from 4 to 27 weeks of age and were exposed to UVB irradiation of the dorsal skin from 7 to 27 weeks of age. The shape, size, and number of skin tumors were examined macroscopically and recorded on a weekly basis. All experimental procedures and protocols were approved by the Animal Experimentation Committee of Chubu University (Approval No. 2410011 and approval date is 12 March 2024) and complied with the National Institutes of Health (NIH) Guidelines for the Care and Use of Laboratory Animals.

### 4.2. Administration of H_2_ and UVB Irradiation

Mice were assigned to either the H_2_ group or the control group. All groups were maintained under identical environmental conditions, and experimental procedures were performed at consistent times to minimize circadian effects. The H_2_ group was treated with both 2% hydrogen gas inhalation and ad libitum access to HRW, whereas the control group received air and dehydrogenated water. Because orally administered H_2_ is rapidly cleared from the body and its biological effects depend on both the dose regimen and administration route [44], we employed a combined protocol of continuous 2% hydrogen gas inhalation and ad libitum access to HRW. This approach was designed to maintain steady-state systemic H_2_ levels via inhalation while providing intermittent bolus exposure through drinking, a pattern reported to produce more pronounced biological effects than those obtained with continuous inhalation alone in other in vivo models [41,42,43]. Hydrogen gas was administered as H_2_-containing air in an acrylic chamber following a previously established protocol [44]. Briefly, mice in standard plastic cages were placed in a sealed 60 L acrylic chamber continuously supplied with a 2% hydrogen gas/98% air mixture at 10 L/min. The gas mixture was generated by combining 100% hydrogen gas (Taiyo Nippon Sanso, Tokyo, Japan) with compressed air from a scroll compressor (SLP-15EB; Anest Iwata, Yokohama, Japan), which was regulated by a multi-flowmeter (Model-1203; Kofloc, Kyoto, Japan). Control animals were housed under identical conditions but received air without hydrogen gas at the same flow rate. Air samples from the chamber were periodically collected and H_2_ concentrations were monitored with an Optical Gas Monitor Model FI-21 (Riken Keiki, Tokyo, Japan) as previously described [44]. Control mice were kept under identical conditions, but breathed air. To ensure consistent H_2_ exposure, HRW was prepared daily (except on weekends) using an Aquela Hydrogen Water 7.0 (donated by MiZ Co., Ltd., Kamakura, Japan). Upon preparation, the dissolved H_2_ concentration (≥0.8 mM) was confirmed using a hydrogen electrode (ABLE, Tokyo, Japan), and bottles were replaced every 24 h to minimize loss of dissolved H_2_. Dehydrogenated water was produced by leaving the HRW open at 4 °C for 48 h.

Mice were exposed to UVB irradiation of the dorsal skin using two G8T5E UVB lamps (SANKYO DENKI, Hiratsuka, Japan) at a dose of 270 mJ/cm^2^ three times per week for 20 weeks from 7 to 27 weeks of age. The UVB irradiation dose influences both the severity of skin damage and the rate of tumor development [76]. Based on established photocarcinogenesis protocols in hairless mice, we selected a dose of 270 mJ/cm^2^ per irradiation. Epstein et al. demonstrated effective tumor induction using three weekly UVB irradiations at 270 mJ/cm^2^ [77]. Prior studies have similarly employed doses of 240–250 mJ/cm^2^ three times weekly in skin carcinogenesis models using hairless mice [78,79]. This dose corresponded to 1–2 minimal erythema doses (MED) [78,80], and we confirmed early erythema formation (Figure S1). This regimen was designed to reliably induce UVB-mediated lesions and tumors within the experimental period while adhering to humane endpoint criteria. After a 10-week post-irradiation observation period, the mice were euthanized at 37 weeks of age for histological examination. Mice were monitored at least once weekly for tumor development, body weight, and overall health status. Tumors were not permitted to exceed a maximum diameter of 15 mm; in practice, mice were euthanized when maximum tumor diameters reached approximately 10–15 mm, depending on tumor location and clinical condition. Humane endpoints were defined according to institutional guidelines. Euthanasia was performed when any of the following criteria were met: body weight loss exceeding 20% from baseline, tumors exhibiting ulceration, necrosis, infection, or persistent bleeding, or tumor growth resulting in impaired ambulation, feeding, drinking, or respiration.

### 4.3. Measurement of CPDs

Genomic DNA was extracted from 5 mm square sections of dorsal mouse skin using the PureLink Genomic DNA Mini Kit (Invitrogen, Waltham, MA, USA). CPD levels were quantified using a High-Sensitivity CPD ELISA Kit Ver. 2 (Cosmo Bio, Tokyo, Japan) according to the manufacturer’s protocol.

### 4.4. Histological and Immunohistochemical Analysis

Skin or tumor tissues were fixed in 10% neutral-buffered formalin for 48 h, followed by dehydration and paraffin embedding. Sections 4 µm thick were prepared for hematoxylin and eosin (HE) staining and immunohistochemistry. Histopathological diagnoses were determined by consensus between the two pathologists based on HE-stained sections. Immunohistochemical staining was performed according to our established protocol [81] using the following primary antibodies: anti-CD3 (Dako, Carpinteria, CA, USA), anti-Gr-1 (eBioscience, San Diego, CA, USA), anti-Mac-2 (Cedarlane, Ontario, Canada), anti-IL-6 (Novus Biologicals, Centennial, CO, USA), and antibodies against STAT3, Ki-67, PCNA, and Nrf2 (Cell Signaling Technology, Danvers, MA, USA).

### 4.5. RNA Isolation and Reverse Transcription Quantitative PCR (RT-qPCR)

Total RNA was isolated using an RNeasy Mini Kit (Qiagen, Hilden, Germany), and 2 µg of RNA was reverse-transcribed into first-strand cDNA using a Transcriptor First-Strand cDNA Synthesis Kit (Roche, Basel, Switzerland). The qPCR step was performed using a LightCycler instrument with FastStart DNA MasterPLUS SYBR Green I (Roche, Basel, Switzerland). The primers used for amplification were as follows: IL-6 (forward, 5′-TCCCAACAGACCTGTCTATACC-3′; reverse, 5′-CAGAGGAAATTTTCAATAGGCA-3′); IL-1β (forward, 5′-TCCTCTCCAGCCAAGCTTCC-3′; reverse, 5′-TTGATGTGCTGCTGCGAGATT-3′); TNF-α (forward, 5′-AACTTCGGGGTGATCGGTCC-3′; reverse, 5′-GCAAATCGGCTGACGGTGTG-3′); and Ptgs2 (forward, 5′-TTCCAATCCATGTCAAAACCGT-3′; reverse, 5′-GGGGTGGGCTTCAGCAGTAA-3′). All procedures were performed as previously described [44].

### 4.6. Immunoblot Analysis

Protein lysates were analyzed by Western blot as previously described [44]. Membranes were incubated with primary antibodies recognizing both the phosphorylated and total forms of p38 MAPK, SAPK/JNK, ERK1/2, and AKT (Cell Signaling Technology, Beverly, MA, USA). β-Actin (Sigma, St. Louis, MO, USA) served as the loading control. Molecular weight markers were run using the Blue Prestained Protein Standard, Broad Range (11–190 kDa; NEB #P7706S; New England Biolabs, Ipswich, MA, USA). Immunoreactive bands were visualized using ECL Prime Western Blotting Detection Reagent (GE Healthcare, Chicago, IL, USA), imaged with a Fusion Solo S system (Vilber Bio Imaging, Collégien, France), and quantified using the ImageJ software (v. 1.53k).

### 4.7. Quantification of GSH and GSSG in Skin

Reduced and oxidized glutathione concentrations in skin samples were determined using a GSSG/GSH Quantification Kit (Dojindo, Kumamoto, Japan).

### 4.8. Statistical Analysis

All statistical analyses were conducted using GraphPad Prism version 10.6.1 (GraphPad Software, Boston, MA, USA). Differences between groups were analyzed using Student’s t-test, one-way ANOVA with Tukey’s post hoc test, or two-way ANOVA with Šidák’s post hoc test, as appropriate. Categorical variables were compared using chi-square tests. The Kaplan–Meier method was used to estimate tumor incidence and survival probability, with differences assessed by the log-rank test. Cox proportional hazards regression was used to estimate hazard ratios and 95% confidence intervals. Data are expressed as mean ± standard deviation (SD). Statistical significance was defined as a two-sided *p* value < 0.05.

## 5. Conclusions

Continuous administration of H_2_ delayed the early inflammatory phases of UVB-induced skin carcinogenesis in HR-1 hairless mice, with independent cohort validation. While CPD levels remained unchanged, the intervention attenuated cutaneous IL-6/STAT3 and ERK/JNK signaling, preserved the redox balance (increased GSH/GSSG after acute UVB exposure and decreased nuclear Nrf2 during chronic exposure), and suppressed epidermal proliferation and thickness. These results demonstrate that H_2_ modulates tumor promotion rather than initiation under chronic UVB irradiation, which explains the observed delay in papilloma genesis. These findings justify the further investigation of H_2_ as a chemopreventive agent for UV-irradiated skin.

## Figures and Tables

**Table 1:** Pathological analysis of UVB-induced skin tumors with or without H_2_ administration.

H_2_ Treatment			(−)	(+)	*p* Value
Number of mice			14	12	
Total number of tumors			135	161	0.42
Histological types of tumors					
	Benign	Papilloma	44 (32.6%)	46 (28.6%)	
	Precancerous/carcinoma in situ	Actinic keratosis-like lesion	4 (3.0%)	5 (3.1%)	
		Carcinoma in situ	4 (3.0%)	6 (3.7%)	
	Invasive carcinoma	Conventional SCCs	77 (57.0%)	98 (60.9%)	
		Spindle cell carcinoma	6 (4.4%)	6 (3.7%)	0.94
Differentiation					
	Conventional SCCs	Well differentiated	57 (68.7%)	78 (75.0%)	
		Moderately differentiated	18 (21.7%)	14 (13.4%)	
		Poorly differentiated	2 (2.4%)	6 (5.8%)	
	Spindle cell carcinoma		6 (7.2%)	6 (5.8%)	0.33
Invasion depth					
	Dermis		43 (51.8%)	52 (50.0%)	
	Hypodermis		11 (13.3%)	12 (11.5%)	
	Muscle		29 (34.9%)	40 (38.5%)	0.86

Categorical variables were compared using chi-square tests.

## Data Availability

The original contributions presented in this study are included in the article/Supplementary Material. Further inquiries can be directed to the corresponding author.

## Supplementary Materials

The following supporting information can be downloaded at https://www.mdpi.com/article/10.3390/ijms27020635/s1.
