# Supplementary material for: Molecular Hydrogen Attenuates Chronic Inflammation and Delays the Onset of Ultraviolet B-Induced Skin Carcinogenesis in Mice
Source: Int J Mol Sci. 2026 Jan 8;27(2):635. doi: 10.3390/ijms27020635 (PMC12840828; doi:10.3390/ijms27020635)
Supplement: Supplementary file 1 [file ijms-27-00635-s001.zip › ijms-4026538-supplementary.pdf]

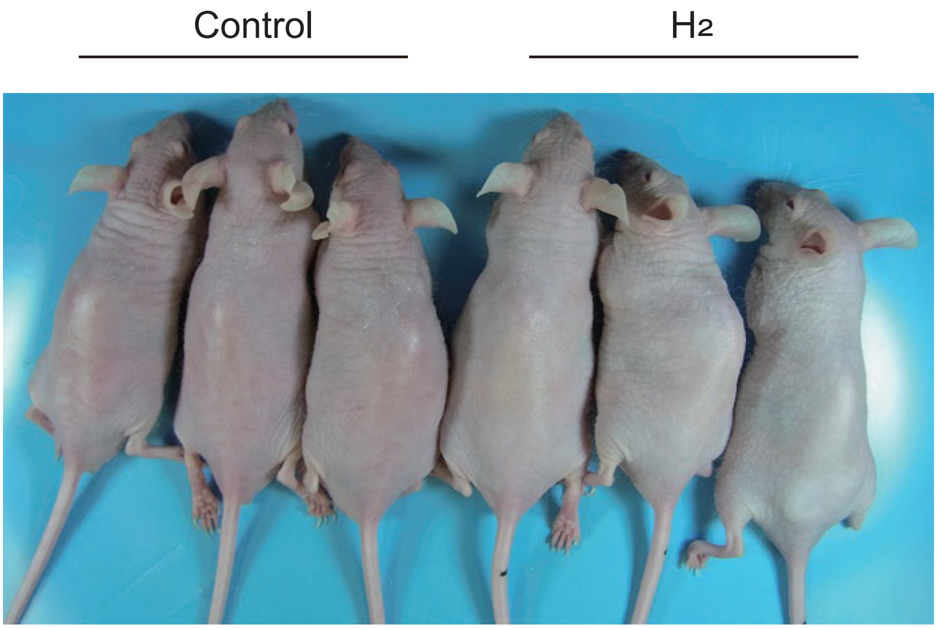

Figure S1. Hydrogen suppresses early-stage erythema induced by UVB irradiation. After 5 weeks of UVB irradiation, dorsal skin photographs show pronounced erythema in controls but considerably milder erythema in hydrogen-treated animals.

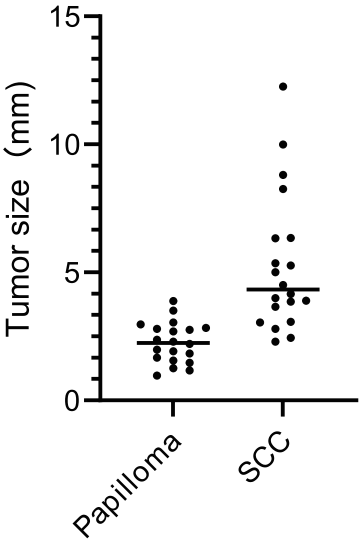

Figure S2. Distribution of tumor sizes in UVB-induced papillomas and SCCs.

Histology and size distribution of skin tumors in mice euthanized 30 weeks after initiating UVB irradiation. SCC: squamous cell carcinoma

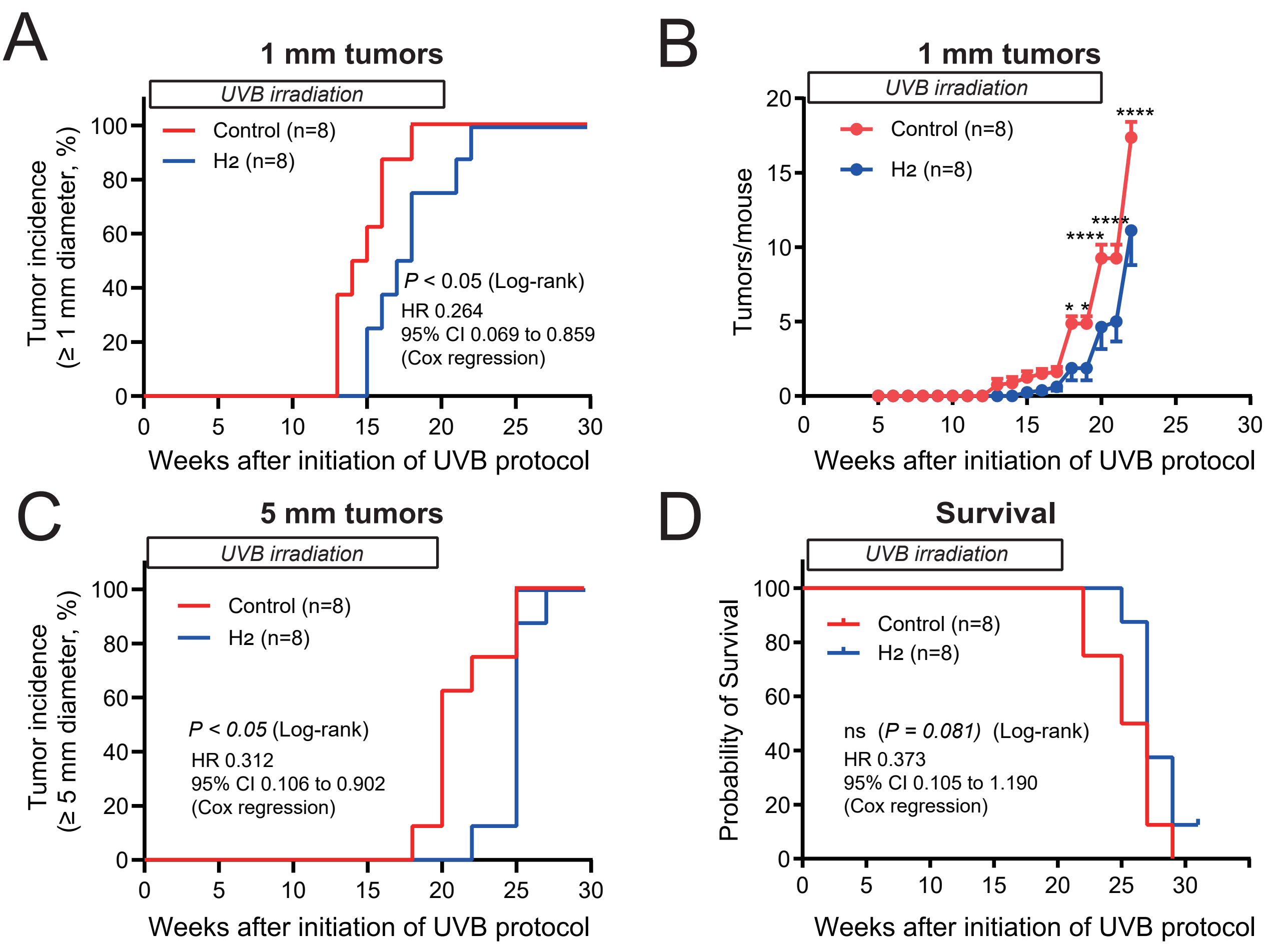

Figure S3. Independent experimental replication confirms the protective effects of hydrogen against UVB-induced tumorigenesis.

(A) Percentage of mice developing tumors (≥ 1 mm diameter) over time following UVB exposure initiation in control (Control) and hydrogen-treated (H2) groups. (B) The total number of tumors per mouse in each group is plotted against the number of weeks since initiation of the UVB exposure protocol. (C) Percentage of mice developing tumors (≥ 5 mm diameter) over time in control and H2 groups. (D) Survival curves comparing Control and H2 groups. Statistical analysis performed using the log-rank test. Results corroborate findings shown in Figure 3. For (A), (C), and (D), the Kaplan–Meier method was used to estimate tumor incidence and survival probability. Differences were assessed by the log-rank test, and Cox proportional hazards regression was used to estimate hazard ratios (HR) and 95% confidence intervals (CI). For (B), differences between groups were analyzed using two-way ANOVA with Šidák's post hoc test. **P < 0.01; ****P < 0.0001.

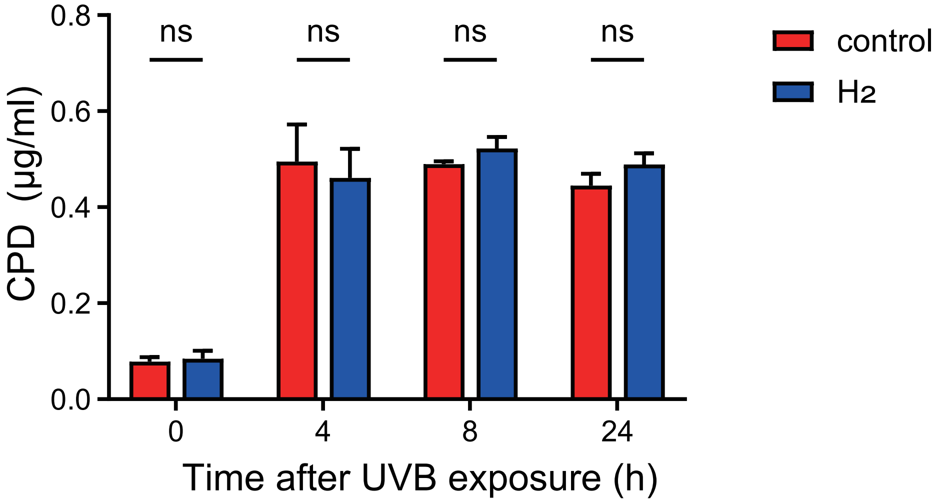

Figure S4. Hydrogen treatment does not influence cyclobutane pyrimidine dimer (CPD) formation.

No differences in CPD levels were observed between experimental groups during the 24-h period following single-dose UVB exposure. Data represent mean ± SD (n = 3). ns indicates not significant.

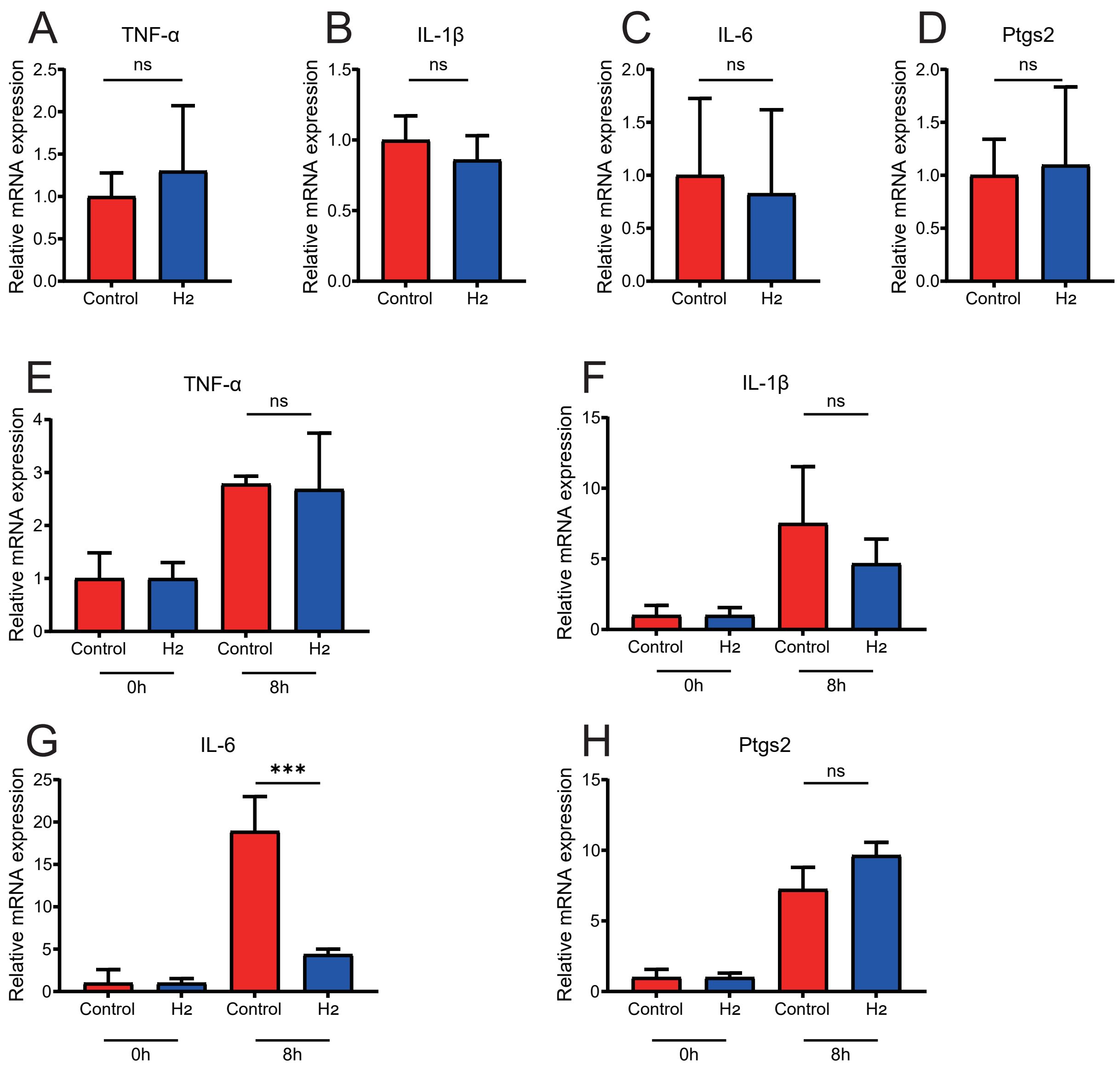

Figure S5. Inflammatory mediator expression remains unchanged during chronic UVB exposure but decreases acutely with hydrogen treatment.

(A–D) Skin mRNA levels of TNF-α, IL-1β, IL-6, and Ptgs2 showed no significant differences between groups after 10 weeks of chronic UVB exposure (n = 5–7). (E–H) Eight hours after a single UVB dose, hydrogen treatment significantly decreased IL-6 mRNA and reduced IL-1β expression, whereas TNF-α and Ptgs2 levels remained similar across groups (n = 3). Data represent mean ± SD. ***$P < 0.001$; ns indicates not significant.
